# Supplementary material for: Randomized controlled trial: Quantifying the impact of disclosing uncertainty on adherence to hypothetical health recommendations
Source: PLoS One. 2022 Dec 14;17(12):e0278263. doi: 10.1371/journal.pone.0278263 (PMC9749967; doi:10.1371/journal.pone.0278263)
Supplement: Attachment — Submitted filename: Response-to-Reviewers.pdf [file pone.0278263.s001.pdf]

Response to Reviewer

2022-07-27

Thank you for your helpful comments, please find a point by point response below.

1. The title of this paper is not appropriate for the content. Consider revising it by colouring it with some specific elements of the research work

We have updated the title to read: Randomized Controlled Trial: Quantifying the Impact of Disclosing Uncertainty on Adherence to Hypothetical Health Recommendations

2. There should be a brief description of how the recruitment of participants was conducted – reproducible explanation.

The section "Participant recruitment and eligibility" covers this topic. We have added details to specify what recruitment via Prolific entails.

3. Conclusion in the abstract should more precise. The present conclusion is vague.

We have updated the conclusion in the abstract to be more specific

4. The conclusion in the body of the paper was not clearly indicated

We have added a conclusion section that reads:

In this randomized controlled trial we observed that disclosing elements of uncertainty in an initial public health statement will change the likelihood that participants will accept new, different advice that arises as more evidence is uncovered. The direction of this effect differed based on whether the participants indicated that they would agree with the final recommendation at baseline. Specifically, among participants who were more likely to agree with the final recommendation at baseline, those who were shown uncertainty initially were less likely to agree with the final recommendation compared to those who were not. Conversely, among participants who were less likely to agree with the final recommendation at baseline, those who were shown uncertainty initially were more likely to agree with the final recommendation compared to those who were not.

5. I consider some of the authorities cited in this work as archaic. It may be more appropriate to cite more current authorities (if available) for issues that are rapidly changing

Thank you, we have added several citations to the introduction in an effort to cite current authorities on the topic.
